# Prevalência de Hipertensão Arterial Sistêmica e Fatores Associados em Indígenas Atendidos em um Ambulatório Especializado no Sul do Brasil

**DOI:** 10.36660/abc.20250269

**Published:** 2025-09-08

**Authors:** Jackson Menezes de Araújo, Leandro Tuzzin, Daniela Teixeira Borges, Jossimara Polettini, Renata dos Santos Rabello, Gustavo Olszanski Acrani, Ivana Loraine Lindemann

**Affiliations:** 1 Universidade Federal da Fronteira Sul Curso de Medicina Passo Fundo RS Brasil Universidade Federal da Fronteira Sul – Curso de Medicina, Passo Fundo, RS – Brasil

**Keywords:** Doença Crônica, Fatores de Risco de Doenças Cardíacas, Pesquisa sobre Serviços de Saúde

## Abstract

**Fundamento:**

A crescente ocorrência de hipertensão arterial sistêmica (HAS) está associada a condições multifatoriais, incluindo mudanças no estilo de vida das populações indígenas.

**Objetivo:**

Estimar a prevalência e verificar os fatores associados à HAS em indígenas atendidos em um ambulatório especializado no Sul do Brasil.

**Métodos:**

Estudo transversal, realizado com amostra de indígenas de ambos os sexos e com 20 anos ou mais, cujos dados foram coletados em prontuários médicos. A hipertensão foi definida como variável dependente, a partir do registro do diagnóstico, para a qual foi estimada a prevalência com intervalo de confiança de 95% (IC95%). Analisaram-se como possíveis fatores associados características sociodemográficas, de saúde e comportamentais, através das estimativas das razões de prevalência (RPs) brutas e ajustadas, acompanhadas de seus respectivos IC95%.

**Resultados:**

A amostra foi composta por 570 indígenas, com prevalência de HAS de 26% (IC95 23-30). Os fatores associados foram idade igual ou superior a 60 anos (RP=1,94; IC95 1,22-3,09), ter cônjuge (RP=1,61; IC95 1,04-2,48) e diagnóstico de diabetes mellitus (RP=2,33; IC95 1,48-3,66).

**Conclusão:**

A prevalência de HAS se mostrou elevada, ratificando seu destaque como um problema de saúde pública também na população indígena e evidenciando a necessidade de fortalecer a saúde primária para atuar na prevenção, no diagnóstico, e no manejo adequado.

## Graphical abstract

**Figura Central:**
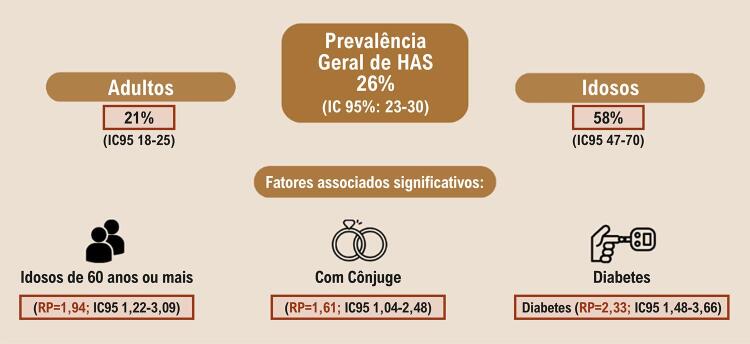
Prevalência de Hipertensão Arterial Sistêmica e Fatores Associados em Indígenas Atendidos em um Ambulatório Especializado no Sul do Brasil

## Introdução

A hipertensão arterial sistêmica (HAS) é uma doença crônica não transmissível (DCNT) que está relacionada a condições multifatoriais.^1^

O diagnóstico em consultório é definido quando a pressão arterial sistólica (PAS) é igual ou maior a 140 mmHg e/ou a pressão arterial diastólica (PAD) é igual ou superior a 90 mmHg. Na medida residencial da pressão arterial (MRPA), a hipertensão é caracterizada por uma PAS igual ou superior a 130 mmHg e/ou a PAD igual ou superior a 80 mmHg.^2^ Essa condição afeta de maneira significativa a qualidade de vida e eleva o risco de doenças cardiovasculares e cerebrovasculares.^3^

Segundo a literatura, a prevalência de HAS entre os indígenas tem aumentado nas últimas décadas. Na década de 1970, a HAS era praticamente inexistente nessa população, com apenas 0,1%, saltando para 7,3% em 1983. Nos anos de 1990, as frequências variaram entre 1,6% e 2,1%, e na década de 2000, os números oscilaram entre 2,0% e 16,3%. Entre 2011 e 2014, a prevalência alcançou 29,7%,^4^ mantendo-se elevada em 2020, com 29,3%. Esse aumento está relacionado a fatores como mudanças no estilo de vida, alimentação e acesso a cuidados de saúde, refletindo a urbanização e a transição epidemiológica.^5^

Observa-se, portanto, que os índices de diagnóstico dessa doença na população indígena têm se aproximado aos da população geral, uma vez que, em 2019, a prevalência da HAS na população mundial geral de 30 a 79 anos era de 33%, enquanto no Brasil era de 41%.^6^

Na América Latina, aproximadamente 55 milhões de indígenas habitam mais de 800 comunidades, correspondendo a 8,5% da população regional, na qual se observa uma mudança demográfica significativa, com cerca de 52% vivendo em áreas urbanas e 48% em áreas rurais.^7^ No Brasil, a população indígena cresceu 88,89% entre 2010 e 2022, totalizando 1 694 836 indivíduos, o que representa 0,83% da população do país. Desses, 54% residem em áreas urbanas, enquanto 46% vivem em zonas rurais.^8^ No Rio Grande do Sul (RS), a população indígena é de 24 958 pessoas, distribuídas por 70 municípios, em 145 aldeias e acampamentos.^9^ Essa transição demográfica tem gerado impactos no perfil de saúde dessas populações, particularmente no aumento da DCNTs, como a hipertensão.

A expansão das fronteiras agrárias, a degradação ambiental, a limitação dos territórios tradicionais, os conflitos fundiários e o aumento do contato com a população não indígena têm contribuído para esse cenário. A proximidade com centros urbanos facilita o acesso a alimentos ultraprocessados ricos em sódio. Além disso, a transformação nas formas de trabalho e a perda de hábitos culturais tradicionais, que incluem a prática de atividades físicas associadas à agricultura e ao cuidado com a terra, também são determinantes nesse processo.^10-12^

Embora o envelhecimento esteja relacionado à presença de HAS, a população indígena no RS apresenta um número reduzido de idosos, com apenas 20,1% tendo 60 anos ou mais. Isso reflete uma qualidade de vida inferior, associada a uma expectativa de vida mais curta.^13^

Em suma, considerando a escassez de estudos focados na investigação da hipertensão em populações indígenas, um grupo que enfrenta desafios específicos quanto ao acesso aos cuidados de saúde, à adaptação de estratégias terapêuticas e à preservação de suas práticas culturais, o presente trabalho objetivou analisar a prevalência de HAS e os fatores associados em indígenas atendidos em um ambulatório especializado no Sul do Brasil. Ao explorar essa questão, busca-se contribuir para uma melhor compreensão do impacto da hipertensão populacional e para o desenvolvimento de estratégias de saúde mais eficazes e culturalmente sensíveis.

## Métodos

O estudo foi realizado com dados originários de uma pesquisa transversal conduzida no Ambulatório de Saúde Indígena mantido pela Universidade Federal da Fronteira Sul em parceria com o Hospital São Vicente de Paulo na cidade de Passo Fundo, no Rio Grande do Sul. A amostra foi selecionada de forma não probabilística, incluindo por conveniência todos os indivíduos com idade ≥ 20 anos, de ambos os sexos, atendidos no ambulatório no período entre 1º de agosto de 2021 e 30 de setembro de 2022.

Passo Fundo, localizada no Planalto Médio do estado, destaca-se como a maior cidade do norte gaúcho, com uma população estimada em 214 564 habitantes. O município abrange uma área territorial de 783 421 Km^2^, resultando em uma densidade demográfica de 262,89 habitantes por Km^2^, distribuídos em 95 659 domicílios. Situada a 289 km da capital Porto Alegre, a cidade possui uma economia baseada na agropecuária e no comércio. Além disso, destaca-se nos setores de saúde e educação superior, sendo reconhecida como o terceiro maior centro médico da região sul do Brasil.^14,15^

O referido ambulatório é o segundo maior do Brasil em atendimentos de alta e média complexidade. Os pacientes são encaminhados ao serviço por meio do Sistema de Gerenciamento de Consultas (Gercon), com o apoio das Secretarias Municipais de Saúde das regiões pertencentes à 6ª, 11ª e 15ª Coordenadorias Regionais de Saúde (CRS), abrangendo 121 municípios.^16^

O protocolo do estudo foi aprovado, com dispensa do Termo de Consentimento Livre e Esclarecido pela Comissão Nacional de Ética em Pesquisa (parecer n.º 5.918.524), obedecendo à Resolução nº 466/2012 do Conselho Nacional de Saúde.^17^

### Análise estatística

A partir da listagem dos pacientes atendidos no período de interesse, foram consultados os prontuários eletrônicos para a coleta de dados. Para atender os objetivos deste estudo, foi analisada como variável dependente o diagnóstico de HAS registrado em prontuário, e como variáveis independentes sexo biológico, idade, condições de moradia, escolaridade, situação conjugal, diagnóstico de diabetes mellitus e de dislipidemia, tabagismo, consumo de bebida alcoólica e prática de atividade física. A análise estatística incluiu a descrição da amostra e a estimativa da prevalência do desfecho (HAS) com um intervalo de confiança de 95% (IC95). Além disso, verificaram-se os fatores associados à HAS por meio da Regressão de Poisson. Na análise bivariada, foram geradas as razões de prevalências (RP) brutas e seus IC95 e, na multivariada, as respectivas medidas de associação ajustadas e seus IC95. Para o ajuste, todas as variáveis de exposição foram inseridas no modelo; aquelas que apresentaram maior valor de p foram retiradas uma a uma para composição do modelo final, em que permaneceram aquelas com valor de p ≤ 0,20. Em todos os testes, admitiu-se um erro α de 5%, sendo considerados significativos valores de p < 0,05, para testes bicaudais.

## Resultados

Na amostra de 570 indígenas, observou-se prevalência de HAS de 26% (IC95 23-30), sendo que em adultos (20-59 anos; n=493) foi de 21% (IC95 18-25) e em idosos (60 anos ou mais; n=76) foi de 58% (IC95 47-70) (Figura Central).

Quanto às características sociodemográficas (Tabela 1), 59,3% eram mulheres e 86,5% adultos; 69,3% residiam em aldeamento; 62,6% cursaram ensino fundamental e 55,4% tinham cônjuge. Em relação à saúde, 11,7% apresentavam diabetes mellitus e 5,4% dislipidemia. Por fim, em relação aos aspectos comportamentais, 26,6% fumavam; 21,6% consumiam bebidas alcoólicas e 7,5% praticavam atividade física.

**Tabela 1:** – Caracterização de uma amostra de pacientes indígenas atendidos em Centro Especializado. Passo Fundo, RS, 2021-2022 (n=570)

Variáveis	n	(%)
**Sexo**		
Feminino	338	59,3
Masculino	232	40,7
**Idade (n= 569)**		
Adultos	493	86,5
Idosos	76	13,4
**Condições de moradia (n= 450)**		
Aldeamento	312	69,3
Acampamento	63	14,0
Outra	75	16,7
**Escolaridade (n= 465)**		
Não alfabetizado	41	8,8
Ensino fundamental	291	62,6
Ensino médio ou mais	133	28,6
**Situação conjugal (n=478)**		
Com cônjuge	265	55,4
Sem cônjuge	213	44,6
**Diabetes mellitus (n= 546)**		
Não	482	88,3
Sim	64	11,7
**Dislipidemia (n= 535)**		
Não	506	94,6
Sim	29	5,4
**Tabagismo (n= 507)**		
Não	372	73,4
Sim/ Ex-tabagista	135	26,6
**Consumo de bebida alcoólica (n= 499)**		
Não	391	78,4
Sim/ Ex-etilista	108	21,6
**Prática de atividade física (n= 464)**		
Não	429	92,5
Sim	35	7,5

No que se refere aos fatores associados à HAS, conforme demonstrado na Tabela 2, foi observada maior prevalência do desfecho nos pacientes indígenas idosos (RP=1,94; IC95 1,22-3,09), entre aqueles com cônjuge (RP=1,61; IC95 1,04-2,48) e com diagnóstico de diabetes mellitus (RP=2,33; IC95 1,48-3,66) (Figura Central). As demais variáveis analisadas não apresentaram associação estatisticamente significativa.

**Tabela 2:** – Fatores de risco associados à hipertensão arterial sistêmica em pacientes indígenas atendidos em Centro Especializado. Passo Fundo, RS, 2021-2022 (n=570)

Variáveis	RP Bruta (IC95%)	p	RP Ajustada (IC95%)	p
**Sexo**		0,242*		0,414*
Masculino	1,00		1,00	
Feminino	1,22 (0,87-1,72)		1,20 (0,77-1,88)	
**Idade**		<0,001*		0,005*
Adultos	1,00		1,00	
Idosos	2,71 (1,90-3,86)		1,94 (1,22-3,09)	
**Condições de Moradia**		0,046^†^		0,555^†^
Aldeamento	1,00		1,00	
Acampamento	1,61 (1,00-2,61)		1,41 (0,75-2,64)	
Outro	1,60 (1,01-2,54)		1,17 (0,60-2,29)	
**Escolaridade**		<0,001^‡^		0,063^‡^
Não alfabetizado	1,00		1,00	
Ensino Fundamental	0,57 (0,35-0,94)		0,88 (0,48-1,60)	
Ensino médio ou mais	0,30 (0,16-0,58)		0,53 (0,25-1,11)	
**Situação conjugal**		0,007*		0,032*
Sem cônjuge	1,00		1,00	
Com cônjuge	1,67 (1,15-2,44)		1,61 (1,04-2,48)	
**Diabetes Mellitus**		<0,001*		<0,001*
Não	1,00		1,00	
Sim	2,93 (2,02-4,23)		2,33 (1,48-3,66)	
**Dislipidemia**		<0,001*		0,578*
Não	1,00		1,00	
Sim	2,56 (1,54-4,25)		1,24 (0,58-2,67)	
**Tabagismo**		0,663*		0,326*
Não	1,00		1,00	
Sim/ Ex-tabagista	0,91 (0,61-1,37)		0,78 (0,47-1,28)	
**Consumo de bebida alcóolica**		0,357*		0,919*
Não	1,00		1,00	
Sim/ Ex-etilista	0,81 (0,51-1,17)		0,96 (0,40-2,26)	
**Prática de atividade física**		0,176*		0,892*
Sim	1,00		1,00	
Não	1,86 (0,76-4,56)		0,90 (0,21-3,96)	

Fonte: Autores.IC95: Intervalo de Confiança de 95%; RP: Razão de Prevalências; * qui-quadrado; † teste de heterogeneidade; ‡ teste de tendência linear. Modelo final composto pelas variáveis idade, escolaridade, situação conjugal e diagnóstico de diabetes mellitus

## Discussão

Neste estudo, a prevalência de HAS apresentou-se menor do que a nacional de 29,3%, conforme os dados da Pesquisa Nacional de Saúde de 2019 sobre a população indígena.^5^ Por outro lado, uma pesquisa internacional com indígenas norte-americanos, que considerou variações populacionais e período recordatório diferentes, apresentou prevalência global inferior à observada no presente trabalho, em torno de 23,5%.^18^

Estudos transversais realizados em diversas regiões do Brasil apresentaram resultados semelhantes aos deste estudo. Como no Parque Indígena do Xingu, localizado no norte do estado do Mato Grosso, com prevalências de 26,7%,^19^ na região oeste do estado do Amazonas, com 29%,^20^ no município de Resplendor, vale do Rio Doce, Minas Gerais, com 31,2%,^21^ e na região do Alto Xingu, situada no nordeste do Mato Grosso, com 37,7%.^22^

No entanto, outras pesquisas, com o mesmo delineamento, identificaram prevalências inferiores às observadas neste estudo, como 20,8% na Reserva Indígena Aracruz, Espírito Santo, localizada na costa sudeste do Brasil,^23^ e 21,8% no município de Campo Novo do Parecis, estado do Mato Grosso.^24^

A diferença nos resultados pode ser explicada pela origem dos dados, pois, no presente estudo, as informações foram extraídas de prontuários médicos, ou seja, de pessoas em acompanhamento nos serviços de saúde. Em contrapartida, algumas das pesquisas mencionadas foram realizadas com relatos autoinformados, enquanto outras realizaram a aferição da pressão arterial apenas no momento de coleta da pesquisa, o que pode subestimar a prevalência real devido à falta de diagnóstico ou ao viés de memória.

A alta prevalência de HAS encontrada entre populações indígenas representa um desafio significativo para a saúde pública. Essa condição é frequentemente assintomática, o que pode levar à subestimação na sua detecção e tratamento precoce. Além disso, as dificuldades no acesso aos serviços de saúde adequados podem exacerbar a doença, contribuindo para maiores complicações cardiovasculares e reduzindo a qualidade de vida dessas comunidades. Esses fatores ressaltam a importância de estratégias de saúde pública externas para o diagnóstico precoce e o manejo adequado da HAS entre populações indígenas.^25,26^

Sabe-se que o envelhecimento está relacionado a alterações fisiopatológicas nos vasos sanguíneos, tais como inflamação, estresse oxidativo, disfunção endotelial e aumento da rigidez vascular, que contribuem para o aumento da pressão arterial.^27^

No presente estudo, a faixa etária mais acometida pela HAS foi a de 60 anos ou mais. A pesquisa em saúde realizada no país em 2019 registrou que a prevalência de hipertensão entre os indígenas idosos (idade ≥ 60 anos) foi aproximadamente 3,6 vezes maior do que na população adulta, com 65% contra 18,1%, (p < 0,001), reforçando a tendência de que, à medida que a idade avança, maiores são os índices de HAS.^5^

Em um estudo transversal realizado no município de Borba, no estado do Amazonas, observou-se que, entre indígenas, a cada um ano adicional de idade, a probabilidade de desenvolver HAS aumenta em 10%, reafirmando essa tendência.^28^

No que se refere à associação do desfecho com a situação conjugal, um estudo transversal prévio realizado no município de Autazes, no estado do Amazonas, revelou prevalência significativamente mais alta entre indígenas sem cônjuge, com 72,7%.^29^ No entanto, os resultados deste trabalho mostraram maior prevalência entre os que possuíam cônjuge. Há evidências de que o casamento pode, de fato, contribuir para o aumento da pressão arterial, visto que os casais têm até 47% mais chances de desenvolver hipertensão juntos, e diante disso, recomendam-se abordagens baseadas em casais para o diagnóstico e o tratamento da HAS.^30^

As divergências nos resultados dos estudos podem ser atribuídas a diferenças contextuais, metodológicas e socioculturais. O primeiro estudo foi realizado em uma comunidade indígena do Amazonas, com estilo de vida, acesso à saúde e papéis de gênero tradicionais distintos em relação ao estudo atual realizado no Rio Grande do Sul. No entanto, destaca-se que relacionamentos conjugais de boa qualidade estão relacionados à diminuição da pressão arterial.^31,32^

O fator de risco de diabetes mellitus é amplamente reconhecido como contribuidor para o desenvolvimento e/ou agravamento da HAS. Sua elevada prevalência nesta amostra reforça essa associação. Os achados estão em consonância com a literatura, pois, no município de Autazes, no estado do Amazonas, no estudo citado anteriormente, observou RP de 3,0 (IC95 1,8-5,1).^29^ Em Dourados, Mato Grosso do Sul, outros pesquisadores encontraram RP de 1,39 (IC95 1,16-1,65).^33^ O controle do diabetes mellitus é essencial para a prevenção e o manejo eficiente da hipertensão, uma vez que esse fator pode aumentar significativamente a carga da doença e impactar negativamente a qualidade de vida dos pacientes.^34^

Portanto, a existência de um centro especializado para populações indígenas é essencial para garantir cuidados de saúde adequados e que respeitem suas especificidades culturais, sociais e linguísticas. Muitos serviços de saúde convencionais não consideram essas particularidades, resultando em tratamentos não efetivos.

A atenção primária à saúde (APS) desempenha um papel fundamental nesse contexto, ao oferecer acesso inicial, contínuo e integral aos cuidados. A APS pode atuar como ponte entre o sistema de saúde e as comunidades indígenas, promovendo ações de prevenção, diagnóstico precoce e manejo de condições crônicas de forma culturalmente sensível.^35-39^

Considerando o exposto, é preciso mencionar que o presente estudo apresenta algumas limitações. A primeira diz respeito ao delineamento transversal, devido à possibilidade de causalidade reversa entre algumas variáveis. Além disso, deve-se considerar o potencial viés de informação, uma vez que a coleta de dados foi realizada com dados secundários. Outro ponto relevante é o possível poder estatístico insuficiente, devido ao tamanho amostral reduzido, na análise referente à identificação dos fatores associados à HAS.

Por fim, o diagnóstico de HAS foi realizado exclusivamente com base nos registros de prontuário. Essa abordagem pode estar sujeita à heterogeneidade nos critérios diagnósticos utilizados durante os atendimentos, além do risco de subdiagnóstico, o que pode impactar a acurácia dos resultados.

Entretanto, o trabalho possui pontos fortes. Destaca-se sua relevância ao analisar dados de um centro de referência e estimular discussões sobre o tema, especialmente considerando a escassez de pesquisas nacionais e internacionais com amostras compostas exclusivamente por populações indígenas, particularmente na região sul do país.

## Conclusão

Os resultados deste estudo, em concordância com a literatura, indicam que a hipertensão é um problema relevante na população indígena, embora sua prevalência possa variar conforme a origem dos dados. A HAS nesse grupo populacional se mostrou associada aos fatores de idade avançada, situação conjugal e diagnóstico de diabetes mellitus. Considerando que a hipertensão é um fator preditivo relevante para complicações de saúde, é fundamental o fortalecimento da APS, com ênfase na prevenção e no manejo adequado da doença. Investir na saúde primária, com foco na hipertensão, representa uma estratégia efetiva para reduzir a carga dessa condição, proporcionando uma saúde integral e acessível a toda a população indígena.
