# Supplementary material for: Prevalência de Hipertensão Arterial Sistêmica e Fatores Associados em Indígenas Atendidos em um Ambulatório Especializado no Sul do Brasil
Source: Arq Bras Cardiol. 2025 Sep 8;122(9):e20250269. [Article in Portuguese] doi: 10.36660/abc.20250269 (PMC12677726; doi:10.36660/abc.20250269)
Supplement: Translation

# Translation: Prevalence of Systemic Arterial Hypertension and Associated Factors in Indigenous Treated at a Specialized Outpatient Clinic in Southern Brazil

**DOI:** 10.36660/abc.20250269i

**Authors:** Jackson Menezes de Araújo, Leandro Tuzzin, Daniela Teixeira Borges, Jossimara Polettini, Renata dos Santos Rabello, Gustavo Olszanski Acrani, Ivana Loraine Lindemann

**Affiliations:** 1 Brasil Universidade Federal da Fronteira Sul – Curso de Medicina, Passo Fundo, RS – Brasil

**Keywords:** Chronic Disease, Heart Disease Risk Factors, Health Services Research

## Abstract

**Background:**

The increasing occurrence of systemic arterial hypertension (SAH) is associated with multifactorial conditions, including lifestyle changes among Indigenous populations.

**Objective:**

To estimate the prevalence and identify factors associated with SAH among Indigenous individuals receiving care at a specialized outpatient clinic in southern Brazil.

**Methods:**

This cross-sectional study was conducted with a sample of Indigenous individuals of both sexes aged 20 years or older, using data collected from medical records. Hypertension was defined as the dependent variable based on the recorded diagnosis, for which prevalence was estimated with a 95% confidence interval (95% CI). Sociodemographic, health-related, and behavioral characteristics were analyzed as potential associated factors through estimates of crude and adjusted prevalence ratios (PR), along with their respective 95% CIs.

**Results:**

The sample consisted of 570 Indigenous individuals, with a SAH prevalence of 26% (95% CI: 23–30). The associated factors were age 60 years or older (PR = 1.94; 95% CI: 1.22–3.09), having a partner (PR = 1.61; 95% CI: 1.04–2.48), and a diagnosis of diabetes mellitus (PR = 2.33; 95% CI: 1.48–3.66).

**Conclusion:**

The prevalence of SAH was found to be high, reinforcing its significance as a public health issue within the Indigenous population as well. These findings underscore the need to strengthen primary health care with an emphasis on prevention, early diagnosis, and proper management

## Introduction

Systemic arterial hypertension (SAH) is a non-communicable chronic disease (NCD) that is associated with multifactorial conditions.^1^

The diagnosis in a clinical setting is defined when systolic blood pressure (SBP) is equal to or greater than 140 mmHg and/or diastolic blood pressure (DBP) is equal to or greater than 90 mmHg. In home blood pressure monitoring (HBPM), hypertension is characterized by an SBP equal to or greater than 130 mmHg and/or a DBP equal to or greater than 80 mmHg.^2^ This condition significantly affects quality of life and increases the risk of cardiovascular and cerebrovascular diseases.^3^

According to the literature, the prevalence of SAH among Indigenous populations has increased in recent decades. In the 1970s, hypertension was virtually nonexistent in this population, with only 0.1%, rising to 7.3% in 1983. In the 1990s, the rates ranged from 1.6% to 2.1%, and in the 2000s, the numbers fluctuated between 2.0% and 16.3%. Between 2011 and 2014, the prevalence reached 29.7%,^4^ remaining high in 2020 at 29.3%. This increase is related to factors such as changes in lifestyle, diet, and access to healthcare, reflecting urbanization and the epidemiological transition.^5^

It is thus observed that the diagnostic rates of this disease in the Indigenous population have come closer to those of the general population, given that, in 2019, the prevalence of hypertension in the global population aged 30 to 79 years was 33%, while in Brazil it was 41%.^6^

In Latin America, approximately 55 million Indigenous people live across more than 800 communities, accounting for 8.5% of the regional population. A significant demographic shift has been observed, with around 52% now living in urban areas and 48% in rural regions.^7^ In Brazil, the Indigenous population grew by 88.89% between 2010 and 2022, reaching a total of 1,694,836 individuals, which represents 0.83% of the country’s population. Of these, 54% live in urban areas, while 46% live in rural zones.^8^ In the state of Rio Grande do Sul, the Indigenous population totals 24,958 people, distributed across 70 municipalities, in 145 villages and settlements.^9^ This demographic transition has impacted the health profile of these populations, particularly with the increase in NCDs, such as hypertension.

The expansion of agricultural frontiers, environmental degradation, the limitation of traditional territories, land conflicts, and increased contact with non-Indigenous populations have all contributed to this scenario. Proximity to urban centers facilitates access to ultra-processed foods high in sodium. In addition, changes in labor practices and the loss of traditional cultural habits – such as physical activities related to agriculture and land stewardship – are also key factors in this process.^10-12^

Although aging is associated with the presence of SAH, the Indigenous population in Rio Grande do Sul has a lower proportion of elderly individuals, with only 20.1% being 60 years old or older. This reflects a lower quality of life, associated with a shorter life expectancy.^13^

In summary, considering the scarcity of studies focused on investigating hypertension among Indigenous populations – a group that faces specific challenges related to access to healthcare, adaptation of therapeutic strategies, and the preservation of their cultural practices – this study aimed to analyze the prevalence of hypertension and its associated factors among Indigenous individuals treated at a specialized outpatient clinic in southern Brazil. By exploring this issue, the study seeks to contribute to a better understanding of the population-wide impact of hypertension and to the development of more effective and culturally sensitive health strategies.

## Methods

The study was conducted using data from a cross-sectional survey carried out at the Indigenous Health Outpatient Clinic maintained by the Federal University of the Southern Frontier (*Universidade Federal da Fronteira Sul*) in partnership with São Vicente de Paulo Hospital in the city of Passo Fundo, in the state of Rio Grande do Sul. The sample was selected non-probabilistically, including for convenience all individuals aged ≥ 20 years, of both sexes, who were seen at the clinic between August 1, 2021, and September 30, 2022.

Passo Fundo, located in the Middle Plateau region of the state, stands out as the largest city in northern Rio Grande do Sul, with an estimated population of 214,564 inhabitants. The municipality covers a territorial area of 783,421 Km^2^, resulting in a population density of 262.89 inhabitants per Km^2^, distributed across 95,659 households. Located 289 Km from the state capital, Porto Alegre, the city has an economy based on agriculture and commerce. In addition, it plays a prominent role in the health and higher education sectors and is recognized as the third largest medical center in southern Brazil.^14,15^

The aforementioned outpatient clinic is the second largest in Brazil for medium- and high-complexity care. Patients are referred to the service through the Consultation Management System (Gercon), with support from the Municipal Health Secretariats of the regions under the 6th, 11th, and 15th regional health coordinators, encompassing 121 municipalities.^16^

The study protocol was approved, with a waiver of the Informed Consent Form, by the National Research Ethics Committee (approval number 5.918.524), in accordance with Resolution No. 466/2012 of the National Health Council.^17^

### Statistical analysis

Based on the list of patients seen during the period of interest, electronic medical records were reviewed to collect data. To meet the objectives of this study, the dependent variable analyzed was the diagnosis of SAH recorded in the medical records. The independent variables included biological sex, age, housing conditions, education level, marital status, diagnosis of diabetes mellitus and dyslipidemia, smoking, alcohol consumption, and physical activity. Statistical analysis included a description of the sample and the estimation of hypertension prevalence with a 95% confidence interval (95% CI). Additionally, factors associated with hypertension were assessed using Poisson regression. In the bivariate analysis, crude Prevalence Ratios (PRs) and their 95% CIs were calculated, and in the multivariate analysis, adjusted association measures and their respective 95% CIs were obtained. For model adjustment, all exposure variables were initially included; those with the highest p-values were removed one by one to build the final model, retaining those with a p-value ≤ 0.20. For all tests, a 5% alpha error was assumed, and values of p < 0.05 (two-tailed) were considered statistically significant.

## Results

In a sample of 570 Indigenous individuals, the prevalence of SAH was 26% (95% CI: 23–30), with a rate of 21% (95% CI: 18–25) among adults (aged 20–59; n = 493), and 58% (95% CI: 47–70) among older adults (aged 60 and over; n = 76) (Central Illustration).

Regarding sociodemographic characteristics (Table 1), 59.3% were women and 86.5% were adults; 69.3% lived in villages; 62.6% had completed elementary school; and 55.4% had a partner. In terms of health status, 11.7% had diabetes mellitus and 5.4% had dyslipidemia. Lastly, regarding behavioral factors, 26.6% were smokers, 21.6% consumed alcoholic beverages, and 7.5% engaged in physical activity

**Table 1:** – Characterization of a sample of Indigenous patients seen at a Specialized Center, Passo Fundo, Brazil, 2021-2022 (n=570)

Variables	n	(%)
**Sex**		
Female	338	59.3
Male	232	40.7
**Age (n= 569)**		
Adults	493	86.5
Elderly	76	13.4
**Housing conditions (n= 450)**		
Villages	312	69.3
Camps	63	14.0
Others	75	16.7
**Education level (n= 465)**		
Illiterate	41	8.8
Elementary school	291	62.6
High school or higher	133	28.6
**Marital status (n=478)**		
With a partner	265	55.4
Without a partner	213	44.6
**Diabetes mellitus (n= 546)**		
No	482	88.3
Yes	64	11.7
**Dyslipidemia (n= 535)**		
No	506	94.6
Yes	29	5.4
**Smoking (n= 507)**		
No	372	73.4
Yes/ Former smoker	135	26.6
**Alcohol consumption (n= 499)**		
No	391	78.4
Yes/ Former alcoholic	108	21.6
**Physical activity (n= 464)**		
No	429	92.5
Yes	35	7.5

Regarding the factors associated with hypertension, as shown in Table 2, a higher prevalence of the outcome was observed among elderly Indigenous patients (PR = 1.94; 95% CI: 1.22–3.09), those with a partner (PR = 1.61; 95% CI: 1.04–2.48), and individuals diagnosed with diabetes mellitus (PR = 2.33; 95% CI: 1.48–3.66) (Central Figure). The other variables analyzed did not show statistically significant associations.

**Table 2:** – Risk factors associated with systemic arterial hypertension in Indigenous patients seen at a Specialized Center, Passo Fundo, Brazil, 2021-2022 (n=570)

Variables	Crude PR (95%CI)	p	Adjusted PR (95%CI)	p
**Sex**		0.242*		0.414*
Male	1.00		1.00	
Female	1.22 (0.87-1.72)		1.20 (0.77-1.88)	
**Age**		<0.001*		0.005*
Adults	1.00		1.00	
Elderly	2.71 (1.90-3.86)		1.94 (1.22-3.09)	
**Housing conditions**		0.046^†^		0.555^†^
Villages	1.00		1.00	
Camps	1.61 (1.00-2.61)		1.41 (0.75-2.64)	
Others	1.60 (1.01-2.54)		1.17 (0.60-2.29)	
**Education level**		<0.001^‡^		0.063^‡^
Illiterate	1.00		1.00	
Elementary school	0.57 (0.35-0.94)		0.88 (0.48-1.60)	
High school or higher	0.30 (0.16-0.58)		0.53 (0.25-1.11)	
**Marital status**		0.007*		0.032*
With partner	1.00		1.00	
Without partner	1.67 (1.15-2.44)		1.61 (1.04-2.48)	
**Diabetes mellitus (n= 546)**		<0.001*		<0.001*
No	1.00		1.00	
Yes	2.93 (2.02-4.23)		2.33 (1.48-3.66)	
**Dyslipidemia (n= 535)**		<0.001*		0.578*
No	1.00		1.00	
Yes	2.56 (1.54-4.25)		1.24 (0.58-2.67)	
**Smoking (n= 507)**		0.663*		0.326*
No	1.00		1.00	
Yes/ Former smoker	0.91 (0.61-1.37)		0.78 (0.47-1.28)	
**Alcohol consumption (n= 499)**		0.357*		0.919*
No	1.00		1.00	
Yes/ Former alcoholic	0.81 (0.51-1.17)		0.96 (0.40-2.26)	
**Physical activity (n= 464)**		0.176*		0.892*
Yes	1.00		1.00	
No	1.86 (0.76-4.56)		0.90 (0.21-3.96)	

95%CI: 95% confidence interval; PR: prevalence ratio; * chi-square test; † test of heterogeneity‡ test of linear trend; final model consisting of the variables age, school attainment, marital status and diagnosis of diabetes mellitus.

## Discussion

In this study, the prevalence of SAH was lower than the national rate of 29.3%, according to data from the 2019 National Health Survey on the Indigenous population.^5^ On the other hand, an international study involving North American Indigenous peoples, which considered different population variations and recall periods, reported an overall prevalence lower than that observed in the present study, around 23.5%.^18^

Cross-sectional studies conducted in various regions of Brazil presented results similar to those of the current study. For instance, in the Xingu Indigenous Park, located in northern Mato Grosso, with a prevalence of 26.7%,^19^ in the western region of the state of Amazonas with 29%,^20^ in the municipality of Resplendor in the Rio Doce Valley, Minas Gerais, with 31.2%,^2 1^ and in the Alto Xingu region, located in northeastern Mato Grosso, with 37.7%.^2 2^

However, other studies with the same design reported lower prevalence rates than those observed in this study, such as 20.8% in the Aracruz Indigenous Reserve, Espírito Santo, located on Brazil’s southeastern coast,^2 3^ and 21.8% in the municipality of Campo Novo do Parecis, in the state of Mato Grosso.^24^

The differences in results may be explained by the data sources, as the present study relied on information extracted from medical records – that is, from individuals receiving follow-up care in health services. In contrast, some of the aforementioned studies were based on self-reported information, while others measured blood pressure only at the time of data collection, which may underestimate the true prevalence due to lack of diagnosis or recall bias.

The high prevalence of SAH found among Indigenous populations represents a significant public health challenge. This condition is often asymptomatic, which can lead to underdiagnosis and delayed treatment. Furthermore, limited access to adequate healthcare services may exacerbate the disease, contributing to more severe cardiovascular complications and reducing the quality of life in these communities. These factors highlight the importance of implementing public health strategies aimed at early diagnosis and proper management of hypertension among Indigenous populations.^25,26^

It is well known that aging is associated with pathophysiological changes in blood vessels, such as inflammation, oxidative stress, endothelial dysfunction, and increased vascular stiffness, all of which contribute to elevated blood pressure.^27^

In the present study, the age group most affected by SAH was individuals aged 60 years or older. The national health survey conducted in 2019 reported that the prevalence of hypertension among elderly Indigenous people (aged ≥ 60) was approximately 3.6 times higher than in the adult population – 65% versus 18.1% (p < 0.001) – reinforcing the trend that hypertension rates increase with advancing age.^5^

In a cross-sectional study conducted in the municipality of Borba, in the state of Amazonas, it was observed that among Indigenous individuals, each additional year of age increased the likelihood of developing HTN by 10%, further confirming this pattern.^28^

Regarding the association between the outcome and marital status, a previous cross-sectional study in the municipality of Autazes, also in the state of Amazonas, revealed a significantly higher prevalence among Indigenous individuals without a partner, at 72.7%.^29^ However, the results of the present study showed a higher prevalence among those who had a partner. There is evidence that marriage may, in fact, contribute to increased blood pressure, as couples are up to 47% more likely to develop SAH together. Consequently, couple-based approaches are recommended for the diagnosis and treatment of hypertension.^30^

The divergences in study results may be attributed to contextual, methodological, and sociocultural differences. The first study was conducted in an Indigenous community in Amazonas, where lifestyle, access to healthcare, and traditional gender roles differ from those observed in the present study conducted in Rio Grande do Sul. Nevertheless, it is worth noting that high-quality marital relationships are associated with lower blood pressure.^31,32^

Diabetes mellitus is widely recognized as a risk factor contributing to the development and/or worsening of SAH. Its high prevalence in this sample reinforces this association. The findings are consistent with the literature; for example, in the municipality of Autazes, in the state of Amazonas, the previously cited study reported a prevalence ratio (PR) of 3.0 (95% CI: 1.8–5.1).^29^ In Dourados, Mato Grosso do Sul, other researchers found a PR of 1.39 (95% CI: 1.16–1.65).^33^ Proper control of diabetes mellitus is essential for the prevention and effective management of hypertension, as this factor can significantly increase disease burden and negatively impact patients’ quality of life.^34^

Therefore, the existence of a specialized center for Indigenous populations is essential to ensure adequate healthcare that respects their cultural, social, and linguistic specificities. Many conventional health services do not account for these particularities, resulting in ineffective.

Primary health care (PHC) plays a fundamental role in this context by providing initial, continuous, and comprehensive access to care. PHC can serve as a bridge between the healthcare system and Indigenous communities, promoting prevention, early diagnosis, and the culturally sensitive management of chronic conditions.^35-39^

Given the above, it is important to note that this study has some limitations. The first relates to its cross-sectional design, due to the possibility of reverse causality between some variables. Additionally, there is a potential for information bias, as data collection was based on secondary sources. Another relevant point is the possible insufficient statistical power due to the reduced sample size in the analysis of factors associated with SAH.

Finally, the diagnosis of SAH was based exclusively on medical record entries. This approach may be subject to heterogeneity in the diagnostic criteria used during consultations, as well as the risk of underdiagnosis, which could affect the accuracy of the results.

Nevertheless, the study presents notable strengths. Its relevance lies in analyzing data from a referral center and fostering discussions on the topic—especially given the scarcity of national and international research with samples composed exclusively of Indigenous populations, particularly in the southern region of the country.

## Conclusion

The results of this study, in line with the literature, indicate that hypertension is a significant health issue among the Indigenous population, although its prevalence may vary depending on the data source. In this population group, hypertension was found to be associated with older age, marital status, and diagnosis of diabetes mellitus. Considering that hypertension is a key predictive factor for health complications, strengthening PHC with an emphasis on prevention and proper disease management is essential. Investing in primary care, with a focus on hypertension, represents an effective strategy to reduce the burden of this condition and to provide comprehensive and accessible healthcare for the entire Indigenous population
